# Osteopathy Again

**Published:** 1900-11

**Authors:** 


### OSTEOPATHY AGAIN.

IT will be remembered that the last session of the State Senate and House of Representatives distinguished itself by passing the "osteopathy bill," which the good sense of the governor turned down by official veto. The newspapers inform us that a strong movement is on foot to push the measure through and give the osteopaths a position equal to that of any graduate of a reputable college who has by the expenditure of much money and diligent labor for four years secured the license to practice medicine. The argument which the osteopaths have advanced is that they do not practice medicine in the sense of giving drugs, and are, therefore, entitled to recognition on their own merits and are not thrown in the same class as the regular medical graduates. But this quibble contains in it the germs of its own destruction, for the legal interpretation of the conception "practice of medicine" is liberal enough to embrace within it much more than the limited claims of the osteopaths. The session of the present house is too young to enable one to judge of its *personnel.* We have only the meager data furnished by the sanguinary exploits of a representative on the free ride to Valdosta, and the notable deed of another who blew out the gas and came in near view of the Elysian fields thereby. If we are to take these two instances to represent the whole, a combination of savagery and bucolic ignorance should be sufficient to give the osteopaths an opening. The character of the local osteopathic "push" renders it difficult to surmise to which element of the legislative body it will attach itself for the fructification of its scheme of recognition—the brutal or the ignorant—for it consists of an anemic Swede of the consultation free variety and a medico-clerical "straddle" who doffs his sacerdotal robes on Monday, after wrestling with dislocated and wandering souls on Sunday, to shove home a few joints which have slyly slipped their moorings in the interim.

If the point of attachment were chosen with an eye to qualification and fitness, it is scarcely necessary to state that the ignorant would be selected.

It may encourage them to add that they will probably gain their purpose, for what are we to expect with a jugulated press and supine officials, and the knowledge behind us that despite the governor's veto, they have been openly plying their trade in our midst for the past year, without let or hindrance.

Perhaps it would be better to have the State law repealed, traduced and ignored as it is, rather than allow it to stand a crumbling ruin that excites only a contemptuous pity and offers an effective defence comparable to the walls of Jericho.

